# Rapid imaging and product screening with low-cost line-field Fourier domain optical coherence tomography

**DOI:** 10.1038/s41598-023-37646-4

**Published:** 2023-07-04

**Authors:** Zijian Zhang, Xingyu Yang, Zhiyi Zhao, Feng Zeng, Sicong Ye, Sara J. Baldock, Hungyen Lin, John G. Hardy, Yalin Zheng, Yaochun Shen

**Affiliations:** 1 Department of Electrical Engineering and Electronics, University of Liverpool, Liverpool, L69 3GJ UK; 2 Department of Eye and Vision Sciences, University of Liverpool, Liverpool, L7 8TX UK; 3 Department of Chemistry, Lancaster University, Lancaster, LA1 4YB UK; 4 School of Engineering, Lancaster University, Lancaster, LA1 4YW UK; 5 Materials Science Institute, Lancaster University, Lancaster, LA1 4YB UK

**Keywords:** Applied optics, Optical techniques

## Abstract

Fourier domain optical coherence tomography (FD-OCT) is a well-established imaging technique that provides high-resolution internal structure images of an object at a fast speed. Modern FD-OCT systems typically operate at speeds of 40,000–100,000 A-scans/s, but are priced at least tens of thousands of pounds. In this study, we demonstrate a line-field FD-OCT (LF-FD-OCT) system that achieves an OCT imaging speed of 100,000 A-scan/s at a hardware cost of thousands of pounds. We demonstrate the potential of LF-FD-OCT for biomedical and industrial imaging applications such as corneas, 3D printed electronics, and printed circuit boards.

## Introduction

Optical Coherence Tomography (OCT) is a non-invasive and non-contact imaging modality that can be thought of as an optical analogue of ultrasound^1^. It is based on the principle of low-coherence optical interferometry for imaging turbid scattering media, which is excellent at rendering depth-resolved images of an object’s internal structure with micron-scale resolution^2^. OCT has undergone tremendous development in the past two decades. With the emergence of Fourier Domain OCT (FD-OCT)^3^, the technology has become indispensable in ophthalmology and branched out into other applications in cardiology, dermatology and gastroenterology^4^, as well as in industrial Non-Destructive Testing (NDT)^5^. The key contributor to the success of FD-OCT has been the Fourier domain detection, enabling an increase in the imaging speed and sensitivity by orders of magnitude than time domain OCT^6^. Despite a superior imaging performance, most of the current commercial FD-OCT systems are priced at tens of thousands thus out of reach for cost-sensitive applications such as for primary care use^7^.

To enhance the accessibility of the technology, efforts have been made to reduce the cost of the first FD-OCT variation, Spectral Domain OCT (SD-OCT), by using inexpensive components and developing cheaper approaches for point-by-point scanning^8–11^. The reported methods involve modifying a commercial spectrometer and using manual scanning techniques^9,12^, or developing customized spectrometers and scanning units that use microelectromechanical (MEMS) mirrors^10,11^. While these low-cost systems have similar image quality to commercial SD-OCT, there is a trade-off between data acquisition speed and line-scan camera cost. Commercial SD-OCT typically operates at a faster rate (e.g., 40,000 A-scan/s) to allow for more useful functions, including lateral repeated scanning and 3D OCT imaging for virtual biopsy^13,14^. A faster FD-OCT technology at a lower cost is desirable for expanding potential applications while retaining the economic benefits of low-cost OCT.

Swept Source OCT (SS-OCT)^15^, as another implementation of FD-OCT, is favored for its fast OCT imaging^16^. SS-OCT uses a high-speed wavelength-swept laser and a dual balanced detector with a high-speed analog–digital converter to record OCT data^17^. Although it can achieve an imaging speed of typical 100,000 A-scans/s, its key components are expensive, and the tunable light source is technologically complex^18^, making it challenging to reduce hardware costs without sacrificing imaging performance. A relatively new technology called Line Field FD-OCT (LF-FD-OCT) has emerged as a fast alternative to traditional SD-OCT^19^. By using parallel illumination and detection with a line-shaped beam, LF-FD-OCT can capture an entire B-scan image in a single shot, which significantly reduces acquisition time compared to sequential A-scan captures. This paradigm shift offers several advantages over traditional SD-OCT, including a reduction in motion-related image distortion and artifacts within a single B-scan measurement, simpler mechanics for 3D imaging, and the ability to reuse the spectrometer configuration in SD-OCT by replacing a 1D line scan camera with a 2D camera (known as the imaging spectrograph)^20–23^. Nevertheless, most of the recent work on developing LF-FD-OCT included mainly ultrafast technology, functional extension and investigation of novel industrial applications^24–27^ and was not aimed at the development of a robust low-cost variant.

In this paper, we report on a high-performance LF-FD-OCT system using cost-effective components. We discuss the selection of key optoelectronic components and describe the system's design, which utilizes a full custom imaging spectrograph and a 2D CMOS camera typically used in mass machine vision applications. By using a single-axis Galvo scanner, which is less expensive than its dual-axis counterpart, we achieved a volume image acquisition rate of 100,000 A-scans/s for 3D OCT data. The resulting LF-FD-OCT system has an axial resolution of 8.3 µm in air, a lateral resolution of 11 µm, and an imaging depth of 2 mm. We demonstrated the 3D visualization of porcine cornea structures and explored the potential industrial applications of this low-cost LF-FD-OCT system.

## Methods

### Imaging spectrograph

**Figure 1:**
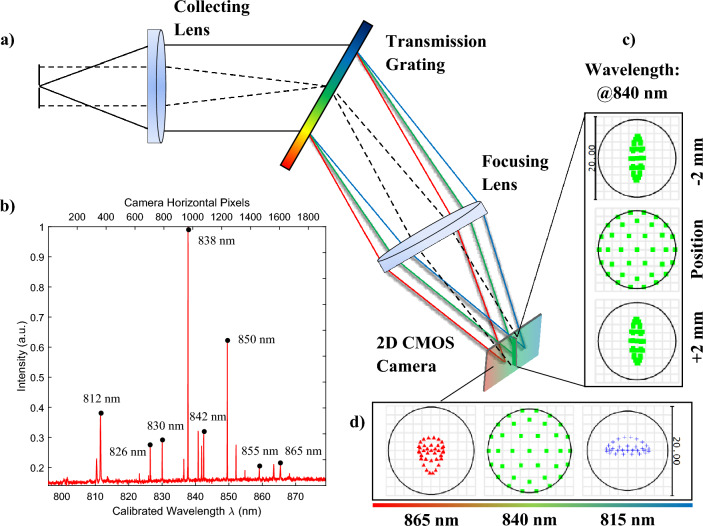
(**a**) Schematic of the imaging spectrograph. (**b**) The calibration with an argon emission source. The wavelengths collected by the horizontal pixels of the spectrograph range from 796 to 879 nm. The FWHM spectral resolution across the wavelength range measured at 812 nm, 826 nm, 830 nm, 838 nm, 842 nm, 850 nm, 855 nm, and 865 nm are 0.18 nm, 0.12 nm, 0.11 nm, 0.08 nm, 0.1 nm, 0.08 nm, 0.1 nm and 0.22 nm, respectively. (**c**) Zemax spot diagram of the 840-nm wavelength along the spatial dimension at the central point and ± 2 mm away from it. (**d**) Zemax spot diagram along the spectral dimension at 815 nm, 840 nm, and 865 nm. In (**c**) and (**d**), the simulated Airy radius is 9.335 µm.

Imaging spectrograph is the core and costly part of a LF-FD-OCT, where a spectrally resolved interference signal of each point on the sample illuminated by the line-shaped beam can be obtained simultaneously^19,28^. This therefore affects the key performance metrics such as axial resolution, imaging depth and imaging speed. Figure 1a shows the custom-designed transmission spectrograph. In the horizontal perspective (solid line in Fig. 1a), the incident light is collimated by an achromatic lens (AC254-100-B, Thorlabs) after passing through a slit (Pyser Optics) and dispersed by a transmission grating (WP-1200/840, Wasatch Photonics). The optical spectrum is then imaged by another identical lens onto the horizontal pixels of a 2D camera (GS3-U3-23S6M, FLIR). In the vertical perspective (dashed line in Fig. 1a), the same optics are used to direct the line-shaped beam that contains the sample’s spatial information to the vertical pixels of the camera. The camera used has been mass produced for machine vision applications, and the sensor (CMOS, IMX174, Sony) contains 1920 × 1200 pixels of 5.86 µm × 5.86 µm. By using an argon emission source to calibrate the spectrograph, the camera detected an 85-nm bandwidth centered at 838 nm in full use of its horizontal pixels along the spectral dimension, shown in Fig. 1b. The spectral resolution measured from a series of wavelengths was close to that targeted of 0.1 nm (see Fig. 1b). During measurement, the spectrograph was able to run at 120 fps (under the 12-bit pixel depth mode of the camera) for parallel B-scan acquisition by global shutter means.

The camera and optics used allow each pixel to sample the spectrum with an interval of 0.04 nm. However, the spectral element determined by its corresponding Airy disk radius was sampled by two pixels of the used camera, resulting in a practical spectral resolution of 0.08 nm. This can be found from the calibrated result at the centre wavelength (see Fig. 1b). Specifically, the implementation of achromatic lenses with a focal length of 100 mm, featuring relatively flatter surfaces, can mitigate spherical aberration along the spatial dimension. This factor is crucial for achieving optimal performance in a LF-FD-OCT spectrograph. Zemax simulations (Fig. 1c,d) show the predicted spot size along both the spatial and spectral dimensions at the camera sensor plane to be below an 8 µm root mean square radius. This straightforward and cost-effective configuration demonstrates the capability to achieve the desired performance, allowing for a typical OCT imaging depth of 2 mm. Additionally, the configuration allows the use of the 2 × 2 pixel binning function of the camera for improved signal-to-noise ratio (SNR) and frame rate, at no cost of the imaging depth.

### Light source and safety

The light source selected for the system is a superluminescent diode (SLD) (EXS210040-01, Exalos). According to the specifications provided by the vendor, the centre wavelength of the SLD can range from 820 to 840 nm, and the full width at half maximum (FWHM) bandwidth can vary between 40 and 50 nm, depending on the current applied to power it. The SLD is driven by a LED driver (LEDD1B, Thorlabs) and mounted on an ESD protection cable (SR9A, Thorlabs) that suites TO-can type laser diode. Being paired with the imaging spectrograph, 85 nm bandwidth of the SLD is covered, corresponding to 13-dB power attenuation of the source. The published work discussed that the use of the non-temperature-controlled SLD source may lead to temperature drifts^29,30^. This could translate into changes in the wavelength and the output power of the SLD source, leading to relative intensity noise (RIN) induced SNR reduction. However, RIN is proportional to the optical power registered per pixel and inversely proportional to the exposure time. As many A-scans are detected in parallel in one B-scan measurement in LF-FD-OCT, the exposure time for each A-scan is increased (e.g., ~ 0.5–1 ms), allowing the suppression of RIN-induced noise. As a precaution, a background spectrum is taken before each experiment and subtracted from the interferogram acquired. In terms of laser safety considerations, the point SLD source is transformed to an anamorphic line focus illumination, and this increases the maximum permitted power for in vivo imaging applications. In contrast, conventional SD-OCT with point focus illumination usually has a much more stringent limit on light power to comply with laser safety regulations^31–33^.

### Scanning optics and speed

LF-FD-OCT technology requires no mechanical scanning for 2D OCT B-scan images. This, in essence, reduces the complexity and the cost of the scanning optics. As a result of parallel detection, only single axis scanning in the direction perpendicular to the illumination line is required to collect 3D OCT datasets. This is realized by a single-axis Galvo scanner system (GVS001, Thorlabs), which allows an acquisition speed of 175 OCT volume scans per second. In reality, the image acquisition speed achieved is 120 B-scans/s mainly limited by the data transfer rate between the camera and the computer. The corresponding image acquisition speed is 100,000 A-scans/s, which is comparable to that of high-end SD-OCT and SS-OCT systems where a high-performance resonant scanner and high-speed detector technology are needed^13^. Although plenty of affordable MEMS scanners are on the market, the mirrors are typically 1–3 mm in diameter, thus limiting the light arriving in the sample when it acts as an aperture stop in our LF-FD-OCT configuration. Another idea is to use a voice-coil mirror scanner to allow a larger clear aperture. However, the off-the-shelf voice-coil mirror scanners are dual-axis architecture and designed to meet ± 25° scan angle, thus over-specified.

### System setup

**Figure 2:**
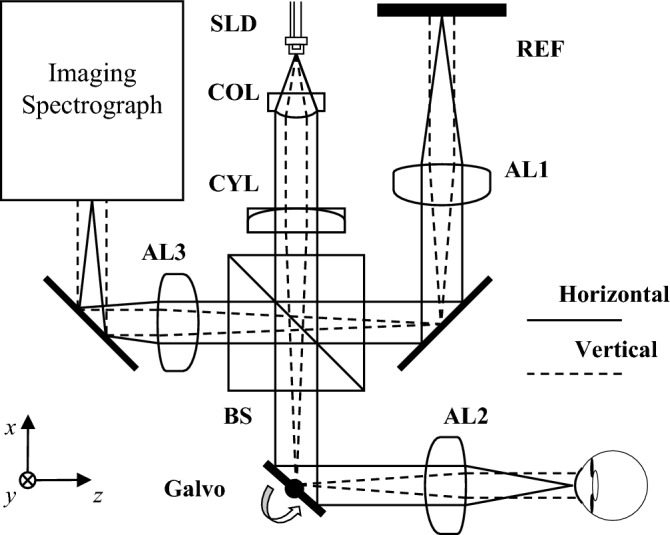
The low-cost LF-FD-OCT system design. SLD: Superluminescent diode; COL: Aspheric collimator lens; CYL: Cylindrical lens; BS: Beamsplitter; Galvo: Single-axis Galvo scanner; AL: Achromatic doublet lens; REF: Reference mirror.

Figure 2 shows the schematic diagram of the low-cost LF-FD-OCT, where the *z*-axis indicates the depth direction of imaging, and the *x*- and *y*- axes correspond to the horizontal and vertical directions in the lateral plane, respectively. The vertical illumination beam has been flipped 90 degrees for display purposes.

The light emitted from the SLD is collimated by an aspheric lens (COL), yielding an astigmatic Gaussian beam to have a 1/*e*^2^ beam width of 4.0 mm vertically and 7.0 mm horizontally. Line shaped illumination is achieved by using a conventional cylindrical lens (CYL), which is split into the two interferometer arms after passing through a non-polarizing 50/50 beamsplitter (BS). The use of the Galvo scanner in the sample arm fanned the probe beam to a sample for volume data acquisition. The reference beam is transmitted to the reference mirror (REF). Two identical objective lenses (AL1 & AL2) were used to focus the horizontal beams, which finally produced a thin illumination line on the sample. The light returned from the two interferometer arms is then recombined and directed to the custom imaging spectrograph.

### Image processing

Given that the autocorrelation signal is negligible in practice, a single point (*x, y*) of the line spectrum registered by the imaging spectrograph can be written as^24^:

1 $${I}_{LF-SDOCT}\left(x,y\right)={I}_{Ref}+{I}_{Sample}\left(y\right)+2\sqrt{{I}_{Ref}{I}_{Sample}(y)}cos\left[2k(x)({Z}_{Ref}-{Z}_{Sample}(y))\right]$$

where the first two terms are direct current (DC) intensities that consist of reflection from reference (${I}_{Ref}$) and sample (${I}_{Sample}$) arms. The third term is the wavenumber-dependent (*k*) interference signal that consists of an intensity factor and a carrier factor with a frequency determined by the optical path difference (OPD) between the reference and sample arms (${z}_{Ref}-{z}_{Sample}$). At each position y, the same wavenumber linearization was employed, and a Fast Fourier transform was then used to reconstruct a B-scan image.

Here, we also present a simple and fast gradient-based segmentation method for automated analysis of the LF-FD-OCT data. The method is a three-step process as follows: (1) Pre-processing: noise artefact removal; (2) Coarse estimation and refinement of air/subject interface; (3) Initial estimation and refinement of other layer interface/s. In the first step, noise and artefacts (horizontal and vertical artefacts) that may affect segmentation performances are detected and removed from the loaded B-scan image. When probe light is perpendicular to the tissue surface, specular reflection is dominant in a narrow region. This leads to a vertical saturation artefact, typically appearing to be a prominent stripe noise in a B-scan image. The saturation artefact is found by looking at the mean intensity of each column (e.g., A-scan waveform) in the image^34^. Horizontal artefacts and vertical artefacts are mitigated and detected by subtracting each row pixel value from the mean intensity of that row and thresholding A-scans above-average intensity, respectively. Noise removal is carried out using a 5 × 5 Wiener de-noise filter. In the second step, the subject surface is coarsely segmented by locating the maximum intensity in each A-scan waveform. The accurate air/subject interface is then determined by maximum gradient summation from the centre to the periphery. All the boundary pixels with decrement weights are used to refine each of the initially estimated interface positions. The pseudo-code for the detailed procedure is shown in Table 1. In the third step, the estimation of other interfaces can be built upon the obtained air/subject interface as prior knowledge due to the correspondence that exists between the layers of many practical samples (e.g., cornea, retina and skin), which can be written as:

2 $$\mathrm{arg}{max}_{\mu \epsilon \alpha }\sum g(y,f\left(y\right)+\mu )$$

and the resultant segmentation is done with the same technique described in step 2.

**Table 1:**
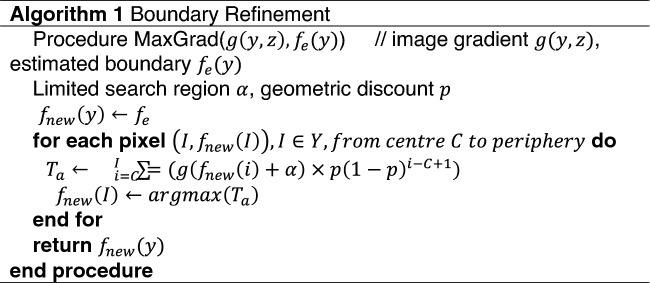
Segmentation Pseudo-code.

### Ex vivo porcine eye imaging study preparation

In the study, the system imaging ability is validated by using ex vivo porcine eye samples. These porcine eye samples are collected from the Morphet & Sons Ltd abattoir in Widnes and are waste products from animals that are entering the food chain. There is a low biological risk of zoonotic infection. The experiment protocols and methods follow the safety regulations of and are approved by the Department of Eye and Vision Sciences and the Department of Electrical Engineering and Electronics at the University of Liverpool.

## Results

### System characterization

**Figure 3:**
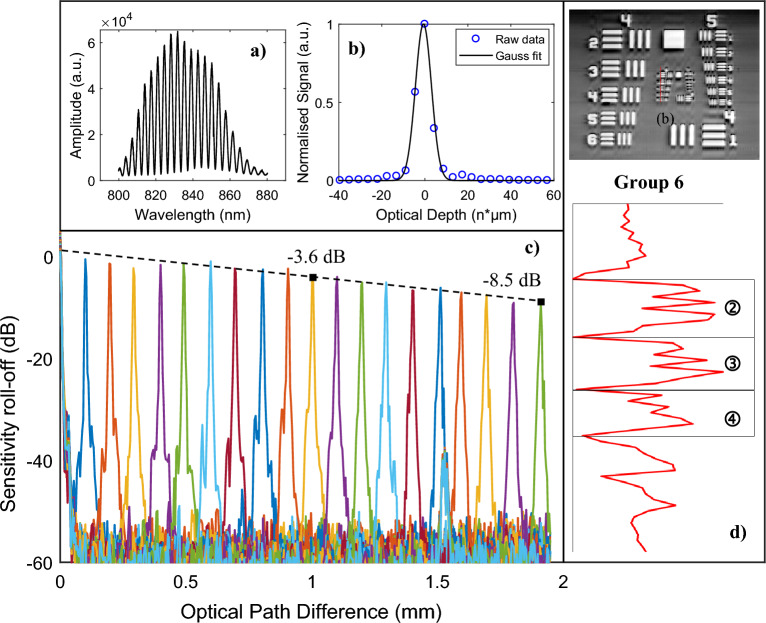
Performance metrics: axial resolution, sensitivity roll-off and lateral resolution. (**a**) A raw interferogram extracted from the acquired spectra of a fixed reflection. (**b**) A Gaussian fit of the zero-padded A-scan, showing an axial resolution of 8.3 μm in air. (**c**) Sensitivity roll-off curve showing an axial range up to 2-mm OPD. (**d**) Lateral resolution measurement using a USAF 1951 (upper) resolution target. Intensity profile (bottom) plotted from the red line position in group 6 demonstrates the smallest resolvable pattern, which is corresponding to element 4 of group 6.

The axial resolution and the sensitivity roll-off of the low-cost LF-FD-OCT system were quantified by measuring a fixed reflection. Figure 3a shows one of the raw interferograms acquired with the imaging spectrograph. It should be pointed out that the current driving the SLD was limited to attain an illumination power of 1.8 mW on the sample, which is well below the accessible emission limit of 9.6 mW calculated for the LF-FD-OCT system^33^. Under this illumination, the SLD's centre wavelength and FWHM bandwidth were measured as 833 nm and 40 nm, respectively. The axial resolution can be determined by analyzing the axial point spread function (PSF), as illustrated in Fig. 3b. By performing a Gaussian fit on the PSF, the axial resolution was measured to be 8.3 μm at the FWHM of the PSF (indicated by the solid line in Fig. 3b). Additionally, the sensitivity of the system was measured to be 85 dB, which is close to the theoretically predicted shot noise limited sensitivity of 90.3 dB. The discrepancy between the experimental and theoretical values could be attributed to the light loss at the slit entrance of the spectrograph. Figure 3c displays the sensitivity roll-off of the system, where roll-off of 3.6 dB and 8.5 dB were measured at positions of 1 mm and 2 mm, respectively, demonstrating the effective imaging depth of the system. The lateral resolution of the system was investigated by scanning a USAF 1951 resolution target. The upper image in Fig. 3d is an OCT *en face* image of the target, and the highest resolution was measured to be 11 µm from the intensity profile through the horizontal elements 2 to 4 in group 6, see the bottom image in Fig. 3d.

**Figure 4:**
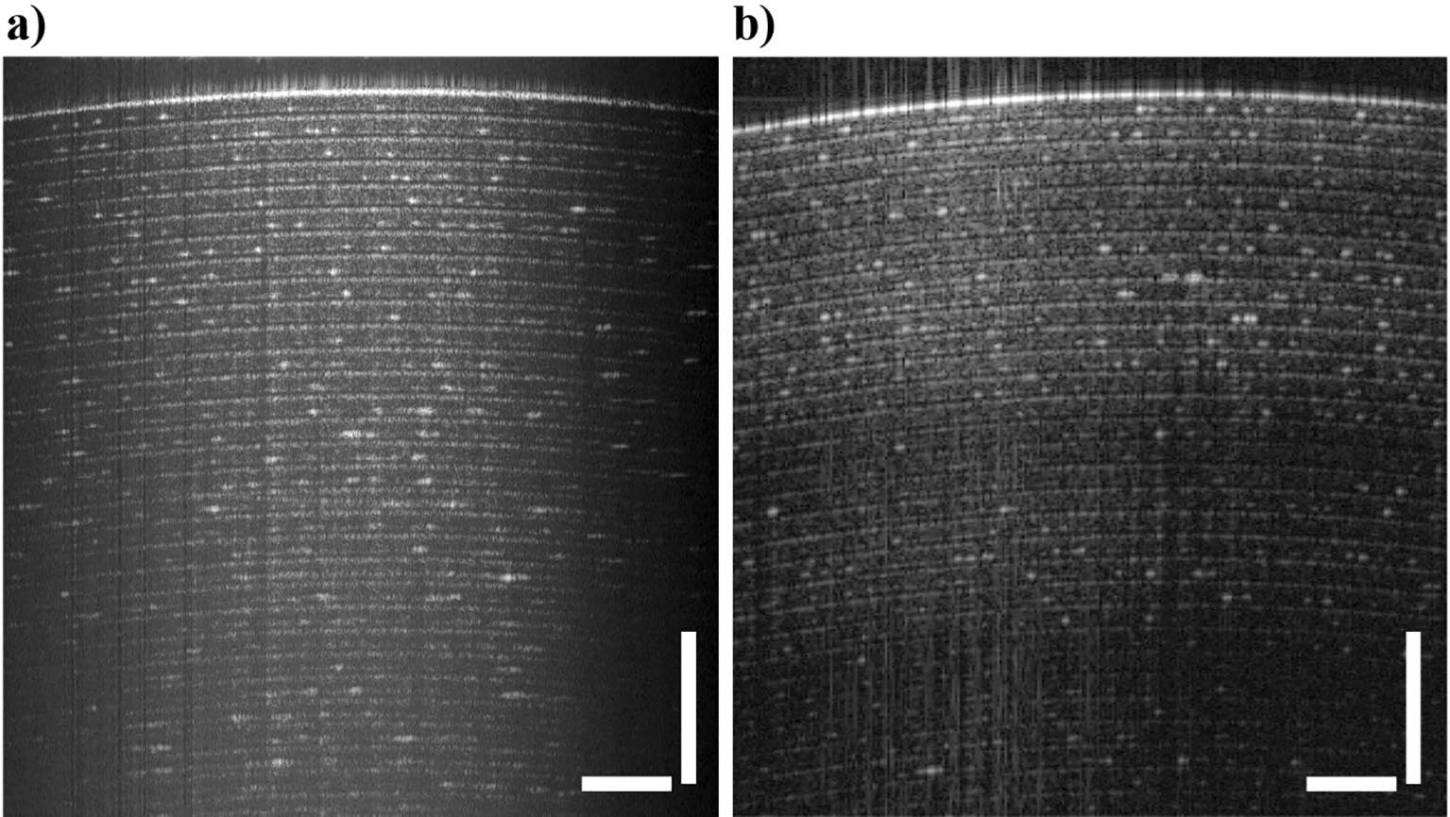
OCT image of a GiftWrap Scotch adhesive tape using (**a**) the low-cost LF-FD-OCT and (**b**) an in-house SD-OCT developed with a commercial spectrometer. The scale bar in each of the images represents 500 µm.

As a further illustration, single-frame B-scan images of a GiftWrap Scotch adhesive tape were acquired respectively by the LF-FD-OCT system (Fig. 4a) and an in-house point scanning SD-OCT system. The SD-OCT was developed by using a commercial spectrometer (Cobra-S 800, Wasatch Photonics) whose spectral resolution is 0.1 nm (Fig. 4b)^35^. Specifically, the power used to illuminate the tape sample in the SD-OCT was 1.4 mW, close to the 1.8 mW power used in the LF-FD-OCT. The integration time used were 10 µs and 500 µs in the SD-OCT and LF-FD-OCT, respectively. It can be found that individual tape layers can be resolved by the LF-FD-OCT from positions slightly better than 2 mm, comparable to the result with the SD-OCT. The reduced contrast observed in the peripheral region can be attributed to the typical use of a cylindrical lens in the LF-FD-OCT setup for creating line illumination. The utilization of a cylindrical lens generates a Gaussian intensity distribution along the line illumination, resulting in a lower signal-to-noise ratio and decreased image contrast in the peripheral region. To address this issue, a Powell lens could be employed^36^. It should be noted that the acquired B-scan data consists of 850 A-scans, covering a vertical region of interest that encompasses 850 out of 1200 pixels along the spatial dimension of the spectrograph. This region is determined by the effective area of line illumination and corresponds to a length of approximately 5 mm when the beam intensity drops to around 5%.

### Ex vivo 3D corneal imaging

**Figure 5:**
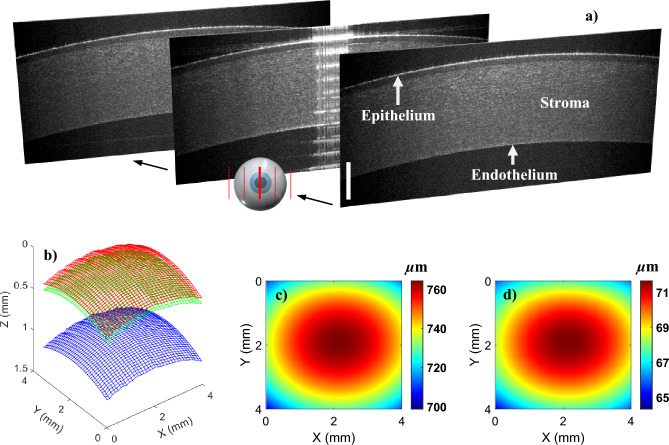
Ex vivo 3D porcine corneal imaging using the low-cost LF-FD-OCT system. (**a**) Representative B-scan images extracted from the acquired volumetric data. The scale bar represents 500 μm. (**b**) Segmented corneal surfaces, including the surfaces of epithelium (red), stroma (green) and endothelium (blue). (**c**) and (**d**) Thickness maps of the total corneal (**c**) and epithelium layer (**d**).

Using the proposed LF-FD-OCT system, we firstly measured a porcine cornea sample ex vivo, in three dimensions. The full 3D data was acquired in 5 s with the 120-fps B-scan rate, and the size of the scanning area is 4 mm × 4 mm in *x–y* plane. Figure 5a shows representative B-scan images along the 1D lateral scanning direction (see inset of Fig. 5a). Structures such as epithelium, stroma and endothelium layers are resolved. Figure 5b shows the segmentation result of the whole cornea region imaged (between red and blue surfaces) and the corneal epithelium layer (between red and green surfaces). The thickness maps are then generated, as shown in Fig. 5c,d, a false color scale is used to map the thickness variation encompassing a range from 700 to 760 µm for the whole corneal region and a range from 65 to 71 µm for the corneal epithelium. As a result, the thicknesses of the structures were calculated to be 743.2 ± 13.1 µm (cornea) and 69.2 ± 1.5 µm (corneal epithelium), which are within the range of reported values^35,37–39^. Notably, the imaged region of interest (ROI) was aligned by manually centering the corneal sample, and it cannot represent the accurate corneal centre.

Corneal pachymetry, the technique of measuring corneal thickness, is of importance in the eye care field, and can aid ophthalmologists in developing treatment plans. OCT is becoming popular over conventional ultrasound pachymetry due to the contactless and high-resolution imaging modality. The efforts put into the development of OCT pachymetry are mainly based upon SD-OCT configuration^40,41^. Few radial scans centered on the measured cornea were acquired to map the corneal thickness. The investigation here illustrates the potential of using an economic OCT system to produce a fine thickness map through a dense 3D OCT scan (e.g., 500 B-scans across a 5-mm cornea area). To meet practical applications, the optimisation of OCT positioning will be carried out in the future, especially for the incorporation of a public-domain pupil tracking technique.

### Potential industrial applications

Due to the absence of mechanical scanning within one B-scan, LF-FD-OCT is less sensitive to environmental influences (e.g., vibration) and is simpler for high-speed imaging applications when compared against SD-OCT. LF-FD-OCTs with these advantages are therefore gaining research interest rapidly in industrial metrology and quality inspection^42–45^. Here we present two short case studies of uses for the proposed system.

#### Measurement of 3D-printed conducting polymers

**Figure 6:**
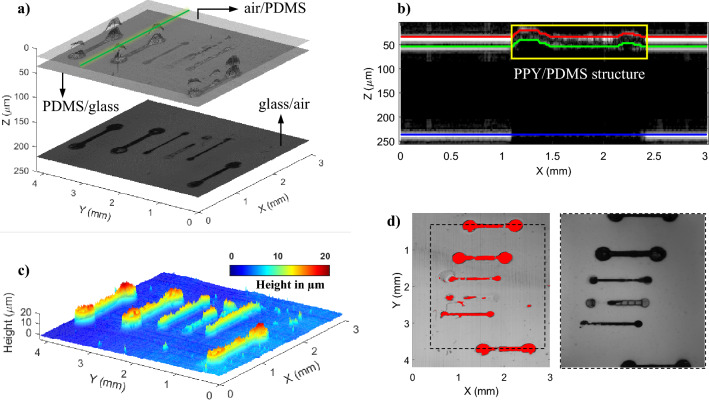
Measurement of 3D printed electrode sample using the low-cost LF-FD-OCT system. (**a**) Volume rendering image of the printed electrode sample. The three interfaces are corresponding to air/PDMS, PDMS/glass, and glass/air interfaces, and the PPY/PDMS structures are within the PDMS layer. (**b**) OCT B-scan image in the *x–z* plane extracted from the position indicated by the green line in (**a**). The area enclosed by the yellow line indicates a cross-sectional PPY/PDMS structure. (**c**) Height profile of isolated PPY structures. The color code displays the varying heights. (**d**) Left: OCT *en face* image in the *x–y* plane with the automatically marked PPY structures in red color; Right: Microscope image of the sample. The area enclosed by dotted lines in the OCT *en face* image represents the same area in the microscope image, which is 2.5 mm × 3 mm (*x–y* plane).

One of the most important innovations over the past years is 3D-printed electronics, which is advantageous over conventional approaches with subtractive manufacturing resulting in a process that is cost-effective and environmentally friendly^46^. So far, time-domain OCT^47^ and SD-OCT^48^ have been used for characterizations and is complementary to 2D optical imaging and surface profilometers. However, the relatively high cost of an OCT is still a barrier for industrial adoption^49^. Here we demonstrate the use of our low-cost LF-FD-OCT system measure 3D-printed electrodes made with conducting polymers^50–52^. The 3D-printed electrode samples were produced using multiphoton fabrication-based rapid prototyping of conducting polypyrrole (PPY) structures within a thin layer of insulating elastomer (polydimethylsiloxane, PDMS) to form six electrodes that were scanned by the LF-FD-OCT to render 3D OCT data. Figure 6a shows the volumetric image of the sample. The area of 3 mm × 4 mm (*x–y* plane) and depth of 250 µm (z) were selected to showcase the whole structure of the sample. As shown in Fig. 6a, three surfaces are distinguished. They are the air/PDMS, PDMS/glass and glass/air interfaces, respectively. And besides, the conducting PPY structures are able to be identified from the changes in signal intensity and then the dumbbell shape imaged. This is contributed by the opaque feature of the PPY polymer, forming a contrast to the transparent PDMS polymer. Figure 6b shows an OCT B-scan image in the *x–z* plane with marked interfaces to include a cross-sectional PPY/PDMS structure, the position of which corresponds to the green line in Fig. 6a. In order to provide more definite structural information, the conducting PPY structures of interest in this study were isolated from the PDMS layer. Figure 6c shows the resulting height profile of the structures. The color code displays the varying heights. The topographic variation is observed to range mainly from 10 to 20 µm. This height information is in addition to the measurement of 2D geometry of the PPY structures by using an OCT *en face* image (see the left image of Fig. 6d).

It is worth underlining that the proposed LF-FD-OCT system demonstrates its ability to image such conducting PPY structures printed within the PDMS base layer, allowing 3D geometry to be measured. This is different from other 2D imaging methods such as microscopy which produces only a bird’s eye view of the sample (see the right image in Fig. 6d). We believe this approach has exciting potential for both rapid prototyping of new structures (e.g., integrated electronics) in academic settings and potentially in quality assurance of additive manufacturing processes applied in industry settings.

#### Rapid inspection of printed circuit board (PCB) coatings

**Figure 7:**
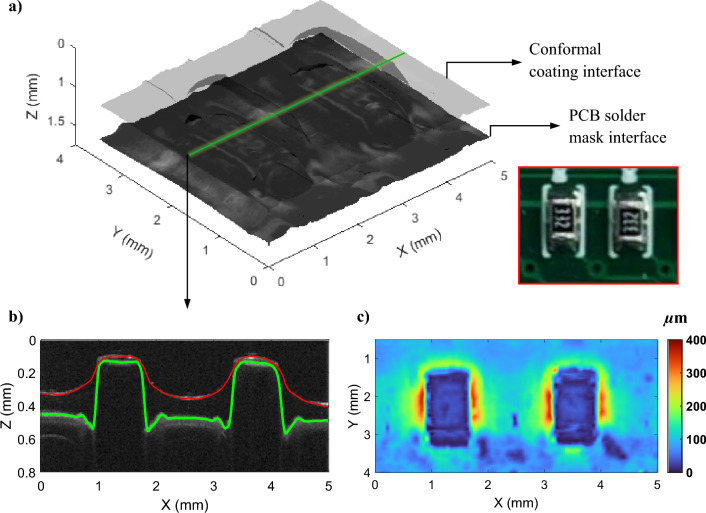
Measurement of printed circuit board (PCB) sample using the low-cost LF-FD-OCT system. (**a**) Volume rendering image of the PCB sample. The two interfaces are corresponding to air/the PCB’s conformal coating and the conformal coating/the PCB’s solder mask interfaces. (**b**) B-scan cross-sectional image in the *x–z* plane extracted from the position indicated by the green line in (**a**). The conformal coating layer is segmented and its upper and bottom boundaries are marked with red and green solid lines. (**c**) Thickness map of the conformal coating.

Conformal coatings have been used for decades to protect printed circuit boards (PCBs) from moisture and corrosion, as well as insulating the underlying ohmic contacts. The prevalent method of coating inspection is sectional imaging under a microscope^52^, which is destructive in nature. Developments in OCT technology have applied SD-OCT and SS-OCT to characterise PCB conformal coatings^53,54^. Here we exploit the low-cost and rapid nature of our system in this field of high-volume manufacturing. In particular, we measured a PCB sample with conformal coating. The PCB is available off-the-shelf and is manufactured by Sci-jet (Mode: SJ-IO-RB24 Monitor). A 3D OCT measurement was made from an area of interest on the PCB that includes two resistors, see the photo inset in Fig. 7a. The size of the measured area is 4 mm × 5 mm in the *x–y* plane. To obtain a detailed insight into the structure of the measured PCB, a 3D image is rendered, as shown in Fig. 7a. In the image, one can distinguish the thin conformal coating layer and the resistors underneath the coating. In addition, one can also observe the topmost solder mask layer (often appearing in green), the function of which is usually the protection of PCB’s copper traces from oxidation. The refractive index mismatch between the conformal coating and solder mask materials allows the interfaces to be resolved by OCT. By using the segmentation algorithm, the conformal coating layer is separated. Figure 7b shows a cross-sectional image with the segmented air/conformal coating interface (the red line in Fig. 7b) and conformal coating/solder mask interface (the red line in Fig. 7b). The position of the image selected corresponds to the green line in Fig. 7a. Furthermore, a thickness map was generated to characterize the conformal coating around the resistors, as illustrated in Fig. 7c. The thickness variation over the imaged PCB area is able to be observed in Fig. 7c. In particular, there are distinctive thickness changes along the edges of the imaged resistors, which is consistent with the B-scan images (Fig. 7b). Also, the coating thickness on the top of the resistors was measured to be much thinner than the area upon the PCB solder mask layer, (e.g., 27.2 ± 7 µm vs. 82.9 ± 13 µm). The presented coating thickness map not only directly reflects the uniformity of the coating but also can be used to identify defects (if exists) in the coating layer.

At a low system cost, the proposed LF-FD-OCT is capable of detecting most of the thin conformal coatings that range from 25 to 200 µm^55^. The system’s high imaging speed (e.g., 120 OCT B-scans/s) may have met the requirement for in-line NDT inspection of PCBs in manufacturing processes, which could be of greater interest to the industry.

## Discussion

We present herein the realisation of a low-cost LF-FD-OCT system and demonstrate its use in the imaging of biological and industrial samples. The total cost of the system, including optics, electronics, and optoelectrical components, is estimated to be around £6,000, as shown in Supplementary Table S1. This cost is notably lower than that of contemporary FD-OCT systems and is comparable to the cost of recently developed low-cost SD-OCT systems^9–11^. Such a LF-FD-OCT could feature an advantageous price and performance ratio, especially when considering an OCT imaging speed at 100,000 A-scans/s which might open up new applications beyond the ones we highlighted here.

In SD-OCT and SS-OCT systems with point-scanning format, high speed 3D imaging requires a fast 2D Galvo scanner (or resonant scanner), whilst SS-OCT systems require an additional fast swept light source. This results in increased costs and engineering challenges for these components. In contrast to SD-OCT and SS-OCT, LF-FD-OCT format scans a sample by a line-focused instead of a point-focused illumination. Owing to this, LF-FD-OCT only needs a 1D Galvo scanner to enable high-speed 3D imaging thus the system is less complex. Despite this, LF-FD-OCT has not been extensively studied in OCT research and has only recently been considered as a commercial format, as its alternatives, SD-OCT and SS-OCT have gained significant academic and commercial popularity in the past two decades. One reason may be that the rejection of out-of-focus signals by parallel LF-FD-OCT is less comparable to SD-OCT and SS-OCT due to the absence of half of the confocal gating. This limitation gives rise to concerns regarding crosstalk issues in the imaging of turbid samples. The presence of a significant level of crosstalk can result in the emergence of ghost scattering signals in the final OCT image. We noted that no significant crosstalk effects were evident from published literature^19–26,56^. Nevertheless, there is a need of a thorough study of the effect of cross-talk on the LF-FD-OCT's performance. In addition, all A-scans in an entire B-scan image are captured simultaneously in a single exposure fashion thus the motion-artefacts within a B-scan are minimum. However, all A-scans are acquired in parallel, and their exposure time is usually longer than the exposure time used in SD-OCT devices thus there were concerns on the washout of the interference fringe caused by the motion in the axial direction during the integration time. Nevertheless, clinical studies conducted using LF-FD-OCT systems by us and other groups have concluded that an integration time of less than 0.5 ms is considered acceptable for in vivo imaging^22,56^. The LF-FD-OCT format is also overshadowed by its lack of engineering simplicity. This is due to the fact that the construction of a LF-FD-OCT system relies on free space optics. By contrast, SD-OCT and SS-OCT can use fiber optics, making system alignment and maintenance easier and enhancing system flexibility and robustness. One possible and cost-effective way to ameliorate this limitation could be to use 3D printing to create a chassis for mounting all the optical components used in LF-FD-OCT.

Previous studies on LF-FD-OCT have placed emphasis on utilizing high-speed 2D cameras in spectrographs to achieve rapid imaging speeds, ranging from a few hundred thousand to megahertz A-scan capture speed^22–26^. These cameras have been available at prices ranging from thousands to tens of thousands of pounds. Additionally, supercontinuum (SC) sources have gained popularity in recent LF-FD-OCT systems due to their ability to achieve axial resolutions that are challenging to attain with SLD sources^23,26^. As a result, it has become common to compare these systems to point-scanning SS-OCT systems within a similar cost range, rather than the more affordable and widely used SD-OCT systems that share similarities in system configuration with LF-FD-OCT (e.g., SLD light source and spectrometer/spectrograph design). This perpetuates the perception that there is a correlation between imaging speed and system cost, wherein a one-order increase in imaging speed typically results in a one-order increase in system cost. Table 2 provides a comparison between low-cost LF-FD-OCT and representative LF-FD-OCT systems, considering imaging performance parameters associated with the adopted camera and light source. It is evident that the low-cost LF-FD-OCT system, while demonstrating "entry-level" performance in terms of resolution and achievable speed, has significantly reduced the overall system cost.

**Table 2:** Specs of the low-cost LF-FD-OCT and reported LF-FD-OCT systems.

	Low-cost LF-FD-OCT	LF-FD-OCT ^22^	LF-FD-OCT ^23^	LF-FD-OCT ^26^
**Light source**				
Type	SLD	SLD	SC	SC
Estimated cost	~ £300	> 2,000	> £10,000	> £10,000
Wavelength	833 nm @ 40 nm (FWHM)	840 nm @ 50 nm (FWHM)	700 nm–1000 nm	950 nm–N/A^a^
Axial resolution	8.3 μm	10.2 μm	2.8 μm	6.0 μm
Power of illumination	1.8 mW	9.7 mW	6.8 mW	126 mW
Integration time	0.5 ms	0.3 ms	5 ms	N/A^a^
**Camera**				
Model	FLIR GS3-U3-23S6M	Atmel ATMOS1M60	Andor Neo 5.5	Phantom v2512
Estimated cost	~ £900	> £2,000	> £10,000	> £80,000
Full well capacity	30,000 e-	65,535 e-	30,000 e-	N/A
Sensitivity	85 dB	89.4 dB	85 dB	102 dB
Frame rate	120 fps	201 fps	98.8 fps	25,000 fps
A-scan rate	100,000 A-scans/s	51,500 A-scans/s	213,000 A-scans/s	11,500,000 A-scans/s

^a^ N/A indicates parameter was not available.

In terms of affordable and reliable FD-OCT variants, the barrier to SD-OCT has now been overcome through system-level low-cost design^9–11^. Specifically, the low-cost SD-OCT technology developed by the group from Duke University has successfully entered the market^10,11^. Despite the imperfections previously discussed in LF-FD-OCT format, the proposed low-cost LF-FD-OCT demonstrates comparable imaging performance and cost compared to its low-cost SD-OCT counterparts, while achieving image acquisition speeds ten times faster. Detailed specifications outlining the imaging performance and corresponding system costs can be found in Table 3. It is worth mentioning that in certain applications where only OCT B-scan images are required, the proposed LF-FD-OCT system provides a potential option for simplification. By eliminating the need for its scanning unit (used for 3D imaging), such as the Galvo scanner and its controller, there is a possibility of reducing component expenses by approximately £2,000. This scan-free version can be particularly advantageous for in-line quality inspection on production lines, where harsh environments may pose a risk of damaging scanning mechanisms^57^.

**Table 3:** Specs of the low-cost LF-FD-OCT and reported low-cost SD-OCT systems.

	Low-cost LF-FD-OCT	Low-cost SD-OCT^9^	Low-cost SD-OCT^10^	Low-cost SD-OCT^11^
Light source	SLD	SLD	SLD	SLD
Centre wavelength	833 nm	840 nm	830 nm	830 nm
FWHM bandwidth	40 nm	50 nm	45 nm	42 nm
Power of illumination	1.8 mW	~ 1.3 mW	0.7 mW	0.68 mW
B-scan range	4.0 mm	Flexible^a^	7.0 mm	6.6 mm
Axial resolution (in air)	8.3 μm	8.1 μm	7.0 μm	8.0 μm
Lateral resolution (in air)	11.0 μm	21.4 μm	17.6 μm	19.6 μm
Imaging depth (in air)	2.0 mm	2.7 mm	2.8 mm	2.7 mm
A-scan rate	100,000 A-scans/s	10,000 A-scans/s	8,800 A-scans/s	12,500 A-scans/s
Sensitivity	85 dB	98.89 dB	99.4 dB	104 dB
System cost	£6,122	~ £5,900	~ £5,800	~ £4,100

^a ^The reported system enables manual and arbitrary control of the scan range.

Our concern regarding the low-cost LF-FD-OCT lies in its fixed B-scan range of 4 or 5 mm, which is determined by the length of the illumination line. This range may not be sufficiently large for certain clinical applications that require imaging of the entire cornea, for instance. One approach to address this limitation is to expand the beam further, thereby extending the length of the line illumination. Another option is to utilize a 2D Galvo scanner to achieve a larger field of view. Nevertheless, it is worth noting that point-scanning SD-OCT offers greater flexibility compared to LF-FD-OCT. For example, it can easily perform radial scans, which is not as straightforward with LF-FD-OCT. In summary, we anticipate that these discussions will assist potential users in selecting an OCT technique that is well-suited for their specific applications.

## Conclusion

In this work, we demonstrated a low-cost LF-FD-OCT system that achieves a speed of 100,000 A-scan/s, and the cost of the system is an order of magnitude lower than that of high-speed commercial OCT systems. The design, selection of main components, and key OCT performance metrics have been elucidated. The advantage of our proposed system is that it allows capturing B-scan images in parallel means, which is differentiated from other developed low-cost SD-OCT systems. We have further demonstrated system's potential for medical and industrial applications in the 3D imaging of porcine cornea, 3D-printed structures in flexible electronics and functional coatings in PCBs. Our proposed system therefore enhances the accessibility of the OCT technology while opening up the possibility to screen products rapidly without a loss of performance.

## Supplementary Information

Supplementary Table S1.

## Data Availability

The data that support the findings of this study are available upon reasonable request from the corresponding authors.
