# Supplementary material for: Rapid imaging and product screening with low-cost line-field Fourier domain optical coherence tomography
Source: Sci Rep. 2023 Jul 4;13:10809. doi: 10.1038/s41598-023-37646-4 (PMC10319780; doi:10.1038/s41598-023-37646-4)
Supplement: Supplementary file 1 — Supplementary Table S1. [file 41598_2023_37646_MOESM1_ESM.docx]

Supplementary Table S1: Component cost of the low-cost LF-FD-OCT system^a^

| **Component** | **Vendor** | **Price Pack** |
| --- | --- | --- |
| **Light Source** |  |  |
| SLED | Exalos | £269.00 |
| Collimator | Thorlabs | £100.64 |
| LED driver | Thorlabs | £228.74 |
| **Subtotal** |  | **£598.38** |
| **Imaging Spectrograph** |  |  |
| Slit | Pyser Optics | £184.96 |
| Grating | Wasatch Photonics | £782.00 |
| Camera | FLIR | £829.00 |
| Lenses | Thorlabs | £141.64 |
| **Subtotal** |  | **£1937.6** |
| **Scanner** |  |  |
| 1D Galvo system | Thorlabs | £773.45 |
| Linear power supplies | Thorlabs | £392.53 |
| Data acquisition card | National Instruments | £380.00 |
| **Subtotal** |  | **£1545.98** |
| **Optics and Electronics** |  |  |
| Beam splitter | Thorlabs | £217.26 |
| Lenses | Thorlabs | £338.26 |
| Mounts and other development kits | Thorlabs | £684.55 |
| PC and development software | HP | £800.00 |
| **Subtotal** |  | **£2040.07** |
| **Total (including taxes)** |  | **£6122.03** |

^a^ component prices from 2019-2020 purchases.
